# Mitochondrial Genome Analysis of Primary Open Angle Glaucoma Patients

**DOI:** 10.1371/journal.pone.0070760

**Published:** 2013-08-05

**Authors:** Deblina Banerjee, Antara Banerjee, Suddhasil Mookherjee, Mansi Vishal, Arijit Mukhopadhyay, Abhijit Sen, Analabha Basu, Kunal Ray

**Affiliations:** 1 Molecular & Human Genetics Division, CSIR-Indian Institute of Chemical Biology, Kolkata, India; 2 CSIR-Institute of Genomics and Integrative Biology, Delhi, India; 3 Dristi Pradip, Kolkata, India; 4 National Institute of Biomedical Genomics, Kalyani, India; Centre for Eye Research Australia, Australia

## Abstract

Primary open angle glaucoma (POAG) is a multi-factorial optic disc neuropathy characterized by accelerating damage of the retinal ganglion cells and atrophy of the optic nerve head. The vulnerability of the optic nerve damage leading to POAG has been postulated to result from oxidative stress and mitochondrial dysfunction. In this study, we investigated the possible involvement of the mitochondrial genomic variants in 101 patients and 71 controls by direct sequencing of the entire mitochondrial genome. The number of variable positions in the mtDNA with respect to the revised Cambridge Reference Sequence (rCRS), have been designated “Segregating Sites”. The segregating sites present only in the patients or controls have been designated “Unique Segregating Sites (USS)”. The population mutation rate (*θ = 4N_e_μ*) as estimated by Watterson’s θ (θ_w_), considering only the USS, was significantly higher among the patients (p = 9.8×10^−15^) compared to controls. The difference in θ_w_ and the number of USS were more pronounced when restricted to the coding region (p<1.31×10^−21^ and p = 0.006607, respectively). Further analysis of the region revealed non-synonymous variations were significantly higher in Complex I among the patients (p = 0.0053). Similar trends were retained when USS was considered only within complex I (frequency 0.49 vs 0.31 with p<0.0001 and mutation rate p-value <1.49×10^−43^) and *ND5* within its gene cluster (frequency 0.47 vs 0.23 with p<0.0001 and mutation rate p-value <4.42×10^−47^). *ND5* is involved in the proton pumping mechanism. Incidentally, glaucomatous trabecular meshwork cells have been reported to be more sensitive to inhibition of complex I activity. Thus mutations in *ND5*, expected to inhibit complex I activity, could lead to generation of oxidative stress and favor glaucomatous condition.

## Introduction

Glaucoma is the second largest blinding disorder after cataract. According to a recent estimate, about 67 million people are visually impaired due to glaucoma, and among them approximately 3.1 million are blind [1]. Among the various subtypes, Primary open-angle glaucoma (POAG) is the most common form of this disease.

The incidence of POAG, primarily an adult onset disease, is positively correlated with age. However, the juvenile form of the disease has also been found in many cases. It is a multi-factorial optic disc neuropathy characterized by accelerating damage of the retinal ganglion cells and atrophy of the optic nerve head [2]. Though its pathophysiology remains elusive, a number of genetic and environmental factors act together to precipitate the disease. To date, 33 loci have been reported to be linked with POAG, however only three genes *viz myocilin (MYOC)* [3], *optinuerin (OPTN)* [4], and *WD Repeat 36 (WDR36)* [5] have been identified by family based studies. Mutations in *neurotrophin 4* (*NTF4)* at GLC1O [6] locus and *ankyrin repeats and suppressor of cytokine signaling box-containing protein 10* (ASB10) at GLC1F locus [7] have also been implicated in POAG in a few cases.

POAG is often associated with elevated intraocular pressure (IOP) caused by the abnormal outflow of aqueous humor through the trabecular meshwork (TM), a meshwork of connective tissue lining the outflow pathway at the iridocorneal angle of the anterior chamber of the eye [8]–[9]. Though IOP reduction is considered to be a potential therapeutic measure in POAG, progression of disease continues even after achieving lower IOP with medication. At the population level, incidence and progression of the disease increases with age even at baseline IOP [10]. This suggests that the vulnerability of the optic nerve gradually increases with aging, which ultimately results in the death of the retinal ganglion cells (RGCs) and degeneration of the optic nerve head [11]. Such pathophysiology has also been observed in aged rodents [12]. To date, no mechanism has been elucidated that explains the relationship between age and neuronal vulnerability to degenerative diseases. However, there is increasing evidence that suggests oxidative stress and mitochondrial dysfunction may play a key role in predisposing neuronal cells to death in age-related neurodegenerative diseases such as glaucoma [13]–[15]. Interestingly, it has been proposed that variations in mitochondrial DNA (mtDNA) and in nuclear DNA genes that encode mitochondrial proteins may lead to aberration in mitochondrial structure and function, thus contributing to POAG pathogenesis [16]. It has also been suggested that mitochondria consume more than 90% of the available free oxygen molecules, 15% of which is converted to reactive oxygen species (ROS) under normal physiological conditions. The mean respiratory activity of mitochondria decreases with age, resulting in higher production of ROS and free radicals [17]. This is supported by the observation that mitochondrial ATP production decreases and ROS increases with age both in humans [18]–[19] and rodents [20]–[21]. Several studies have shown that mitochondrial abnormalities, including defects in oxidative phosphorylation, increased accumulation of mitochondrial DNA defects, impaired calcium influx, accumulation of mutant proteins in mitochondria, and mitochondrial membrane potential dissipation are important cellular changes in both early and late-onset neurodegenerative diseases like Amyotrophic lateral sclerosis, Alzheimer’s disease, and Parkinson’s disease [22]–[23]. A transgenic mouse model bearing a familial Alzheimer’s disease mutation showed mutation-specific alterations in mitochondrial dynamics, morphology and function that preceded the onset of memory and neurological phenotype and the formation of amyloid plaques [24]. Various mutations causing the familial form of Parkinson’s disease have been found to alter multiple aspects of mitochondrial biology, including mitochondrial biogenesis, bioenergetics, dynamics, transport, and quality control [25]. Altered mitochondrial fission and fusion might also play a role, as it controls the structure, morphology and number of mitochondria in a cell [26]–[27]. Therefore, the health and activity of mitochondria are central in the aging process. Nevertheless, uncertainty prevails over the fact - whether or not accumulation of mitochondrial mutations leads to a decline in mitochondrial function.

The proposed mechanism of RGC death through apoptosis in a murine model is similar to other optic neuropathies associated with mitochondrial dysfunctions [28]. A recent study demonstrated that mitochondrial dysfunction and AIF (Apoptosis Inducing Factor) translocation from mitochondria may play crucial roles, both in RGC death and in axonal degeneration, the primary target of IOP elevation in experimental rat glaucoma models [29]. Studies on mice subjected to ocular hypertension have shown COX (Cytochrome oxidase) reduction, mitochondrial fission, and cristae depletion [30]. In addition, an increase in IOP has been correlated with altered OPA1 (optic atrophy 1) expression and induction of OPA1 release, a protein that plays a crucial role in mitochondrial inner membrane fusion [31]. A study reported a spectrum of mitochondrial abnormalities in 27 POAG patients, including a decrease in the mean respiratory activity of mitochondria in patients compared to controls [32]. Another study reported that defects in complex I contributed to progressive loss of TM cells in POAG patients by promoting excessive mitochondrial ROS production and by decreasing mitochondrial membrane potential and ATP synthesis [33]. These events result in accelerated aging of the TM cells in POAG patients, thereby driving the cells towards apoptosis [33]. It has also been found that mtDNA4977 deletion is dramatically higher in POAG patients, and the ratio of mtDNA to nuclear DNA is decreased [34]. In addition, there are reports of involvement of mitochondrial protein-coding genes in Normal Tension Glaucoma [35]–[36]. These findings further substantiate a major role of mitochondrial dysfunction in glaucomatous optic nerve degeneration.

In our cohort, among the known candidate genes for POAG, *MYOC* has been found to contribute to disease pathogenesis in only 3% of the reported cases [37]. On the other hand, although a putative mutation (Arg545Gln) was identified in *OPTN*, the gene was not found to play a significant role in POAG causation [38]. Similarly, analysis of *WDR36* suggested a possible association between a *WDR36* SNP and POAG [39]. In addition, common variants and mutations in *CYP1B1, IL1,* and *OPTC* were also found to be associated with the disease [39]–[42]. Several studies to date have reported structural and functional alterations in mitochondria and their metabolites in the pathogenesis of neurodegenerative diseases. However, the involvement of mtDNA in POAG pathogenesis has only been investigated based on sequencing of targeted regions of the mitochondrial genome in a small number of patients (n = 27) [32]. In this study, we have undertaken mtDNA sequencing in a relatively larger number of patients (n = 101) and controls (n = 71); lacking mutations in the known candidate genes for POAG to evaluate the underlying genetic lesion.

## Materials and Methods

### Ethics Statement

Peripheral blood samples were collected from study individuals with their written consent. The study protocol adhered to the tenets of the Declaration of Helsinki and was approved by the “Human Ethical Committee” of the Council for Scientific and Industrial Research - Indian Institute of Chemical Biology.

### Selection of Study Subjects

POAG patients and control subjects were selected from Dristi Pradip Eye Clinic, Kolkata. All participants of the study were inhabitants of Kolkata, West Bengal (eastern India) and belong to the Indo-European linguistic group [43]. The cohort consisted of 101 POAG patients and 71 controls.

Diagnosis of patients involved clinical, ocular and systemic examinations. Intraocular pressure (IOP) was measured by Goldmann applanation tonometry (Haag-Streit USA Inc., Mason, OH) followed by pachymetry (Ocuscan A, Alcon, Texas, USA). A Goldman 3-mirror gonioscope (Ocular Instrument, Bellevue, WA) was used to assess the angles of the anterior chamber and the optic disc. The optic disc was also evaluated with a +78D lens. Automated threshold field analysis was done using Humphrey Field Analyzer II (Carl Zeiss, Dublin, CA). The retinal nerve fiber layer (RNFL) was investigated by Scanning Laser Polarimetry (SLP) with variable corneal compensation technique (GDx-Vcc/GDx-Pro, Carl Zeiss, Dublin, CA).

The POAG patients were diagnosed by the presence of a clinically open angle (angle of the anterior chamber) on Gonioscopy, and significant cupping of the optic disc (>0.7) with or without peripapillary changes. This was further confirmed by typical reproducible visual field changes, *viz.* Arcuate, Bjerrum, Seidel, paracentral and annular scotoma and nasal steps and SLP for RNFL analysis (Nerve Fiber Indicator>30). The pre-perimetric cases were identified by RNFL analysis. IOP was also examined, and in most cases it was found to be above 21 mm of Hg. Individuals with any history of inflammation, ocular trauma (past and present), high myopia (>8 diopter) and ocular hypertension were excluded from this study. Thus, the patient pool consisted of 101 adult onset open angle glaucoma cases. The age at diagnosis ranged from 38 to 88 years, with a median ± standard deviation (SD) of 60±12 years. The patients were not known to have any other ocular disorder.

In this study, 71 controls were recruited following the criteria which include: age >50 years (median age ± SD, 56±11.1 years) without any family history of glaucoma or ocular hypertension, IOP less than 20 mmHg in both eyes in at least last two checkups, no visual field defect, normal SLP parameters i.e. a good yellowish bow-tie scan pattern, deviation map within normal limit, a good double hump pattern in conduction map, temporal-superior-nasal-inferior-temporal (TSNIT) parameters within normal limit, Nerve Fiber Indicator <30 (for both eyes). Cup to disc ratio considered was <0.5 and similar in both eyes, with no defect in disc rim or margin and no sphincter haemorrhage around the disc. Individuals with high myopia (>8 diopter), diabetes and hypertension were excluded from the control group.

### Collection of Blood Samples and Genomic DNA Preparation

Eight milliliters of peripheral blood was collected with EDTA from POAG patients and normal individuals with their written consent. It was ensured that the individuals were unrelated to at least the first cousin level. Genomic DNA was prepared from fresh whole blood using the PAX gene blood DNA isolation kit (Qiagen, Hilden, Germany) according to manufacturer’s protocol. The DNA was dissolved in TE (10 mM Tris-HCl, 1 mM EDTA, pH 8.0).

### mtDNA Amplification and Sequencing

The entire coding region of the mitochondrial genome was amplified in 24 separate polymerase chain reactions (PCRs), using single-set cycling conditions, for all patients and control subjects. Each successfully amplified fragment was directly sequenced in both forward and reverse directions. This entire work was carried out at The Centre for Genomic Application (TCGA, New Delhi). The list of primers is available on request.

### Analysis of mtDNA Sequence in Patients and Controls

Mitochondrial DNA sequences from both patients and controls were analyzed using multiple alignment tools (DNAstar) [44] and compared with revised Cambridge Reference Sequence (rCRS) (NC_012920) from Mitomap (http://www.mitomap.org) [45] to identify the variants present, if any. All identified variants, whether in patients or controls, were compared with variants listed in the Mitomap database and Medline listed publication to check the novelty of the change. We used Sanger sequencing as the detection method; therefore, low level heteroplasmic changes could not be scored.

The aim of the study was to identify the region in the mitochondrial genome which might relate to the pathogenesis of the disease. For this the variants were divided into three categories: (a) the variants present only in the patients which might be associated with the disease, (b) those present only among the controls, and (c) the polymorphic sites present in both patients and controls. The number of variable positions in the mtDNA with respect to rCRS has been designated “Segregating Sites”. Say among 3 individuals, if we restrict to a certain ‘region’ (e.g. coding, genic, ND5 gene) of the mt genome, the 1^st^ individual has variations at 4 sites (a,b,c, and d), the 2^nd^ individual has variations at 3 sites (a,c, and e), and the 3^rd^ individual has variations at 2 sites (a and e), at overlapping loci; then the total number of segregating sites are 5 (a, b, c, d, e). The segregating sites present only in the patients or controls have been designated “Unique Segregating Sites (USS)”.

Frequencies were calculated as the ratio of the number of variations for a specific region (e.g. coding region/complexes or genes within it, RNA region etc.) to the total number of variations identified comprising that region. For example, in Table S1, under patients, the ratio of the number of changes in coding region (243) to the total number of changes in the mtDNA (i.e 243+39+69 = 351) is the frequency (i.e. 243/351 = 0.69) of segregating sites in the coding region. The other frequencies were also calculated in the same manner. The significance of the difference in frequencies among patients and controls were determined by z-test of proportion.

Watterson theta (*θ_w_ = 4N_e_μ*), which is the product of four times the effective population size (*N_e_*) and the mutation rate (μ), was estimated to determine the population mutation rate, and normalized for the number of individuals [46].

For a sample of n individuals, an unbiased estimate of θ is given by 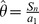 where 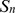
is the number of segregating sites observed and 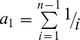.The variance of the estimator is given by 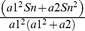 where 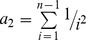.

Watterson has also shown that the distribution of the estimate is asymptotically normal in samples of sufficient size [46]. We utilized this fact to test for significance of the difference in 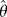 among patients and controls. We assumed asymptotic normality and a possible inequality of variance and applied the Welch’s modification of the t-test for the test of significance [47]. We used the Kolmogrov-Smirnov non-parametric test to compare between the equality of distributions [48]–[49].

All reported and novel mutations detected in mitochondria were analyzed by PolyPhen-2 [Polymorphism Phenotyping v2 (http://genetics.bwh.harvard.edu/pph2/)], a tool that predicts the possible impact of an amino acid substitution on the structure and function of a human protein using straightforward physical and comparative considerations. The output distinguishes the variants into 3 categories: benign, possibly damaging and probably damaging.

## Results

In the present study, we investigated the possible involvement of the mitochondrial genomic variants in a cohort of 101 patients and 71 controls by sequence analysis of the entire mitochondrial genome from peripheral blood. The samples were selected based on prior analysis for absence of mutations in three known nuclear candidate genes (viz. *MYOC*, *OPTN*, and *WDR36*). In the present study, we aim to identify the regions of the mitochondrial genome that show a higher preponderance of changes in a group of POAG patients relative to controls.

### mtDNA Analyses of POAG Patients and Controls

The aim of the study was to identify the region in the mitochondrial genome that might relate to the pathogenesis of the disease. The number of segregating sites unique to patients was found to be 351 in 101 patients compared to 236 in 71 controls. In addition, 284 polymorphic sites were found in both patients and controls. Thus, sequencing the entire mitochondrial genome yielded the presence of 635 segregating sites in 101 patients and 520 in 71 controls. The value of θ_w_ (an estimate of the overall population mutation rate) for the patients was 122.41 (±29.67) compared to 107.59 (±27.86) for controls, indicating that the mutation rate in the patients is indeed higher (p-value <0.00053) than the controls. When only the unique segregating sites (USS) were considered, the estimate of θ_w_ was 67.66 (±16.57) for patients and 48.83 (±12.85) for controls. This difference between patients and controls was even more significant (p = 9.8×10^−15^).

The number of transversion was 26 in patients and 21 in controls. In both patients and controls the ratio of transition to transversion (T_N_/T_V_) was higher in the coding region compared to the control region (Table 1), which attests to the accuracy of the sequencing [50]. We noted a lower ratio of transition to transversion (T_N_/T_V_) in the patients compared to the controls (22 vs 30.60), indicating the preponderance of transversions in patients (Table 1).

**Table 1:** Number of segregating sites, transitions, transversions and T_N_/T_V._

Mitochondrial regions	No of segregating sites		No of transitions (T_N_)		No of transversions (T_V_)		T_N_/T_V_	
	Patients	Controls	Patients	Controls	Patients	Controls	Patients	Controls
**Control Region**	155	144	147	137	8	7	18.38	19.57
**Coding Region**	414	318	396	308	18	10	22	30.8

### Distribution of Segregating Sites in mtDNA

**Figure 1:**
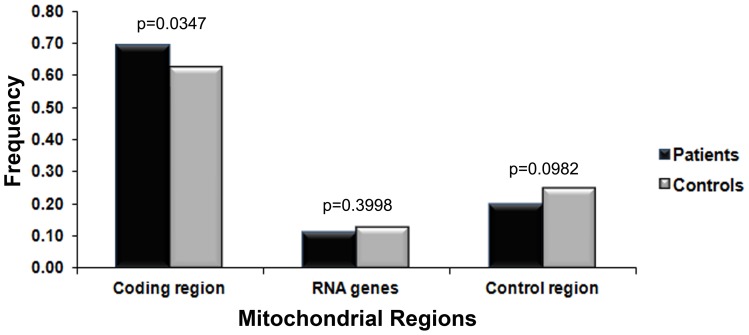
Distribution of segregating sites in different regions of mtDNA. The frequency of segregating sites was marginally higher for coding regions in the patients relative to the controls (0.69 vs 0.62, p = 0.0347). No significant difference was observed in the RNA and the control region.

To evaluate the possible clustering of variants in a specific mitochondrial region, the variants were analyzed in the protein coding regions, tRNA and rRNA coding regions and control regions separately. In the coding region, the frequency of segregating sites was marginally higher in the patients relative to the controls (0.69 vs 0.62, p = 0.0347) (Figure 1 & Table S1). The estimate of θ_w_ was 46.85 (±11.59) for patients and 30.42 (±8.14) for controls. This difference is extremely significant with a p-value <1.04×10^−21^. The θ_w_, which provides the best estimate of the population mutation rate, is marginally higher for patients [13.3 (±3.54)] than controls [12 (±3.42)] (p-value <0.008) for the control region too. No significant difference was observed in either tRNA or rRNA (Figure 1 & Table S1).

### Analysis of Variants in the mtDNA Coding Region

Having observed that the variation in the coding region of mtDNA was significantly higher in the patients compared to the controls (Figure 1), the region was further dissected for each mtDNA complex gene. There was no significant difference in the distribution of segregating sites among patients and controls (Kolmogorov-Smirnov D = 0.1091, p-value = 0.7043) in the 13 protein-coding genes of the mtDNA coding region. However, analysis with only the unique changes in either patients or controls, USS, gave rise to 243 USS in 101 patients compared to 147 in 71 controls (Table 2). The difference in the distribution of USS between the patients and controls was very significant (Kolmogorov-Smirnov D = 0.2617, p-value = 0.006607). The θ_w_ estimate for the coding region, not normalizing for the number of basepairs, is 46.84 for patients compared to 30.42 for controls (p value <1.13×10^−21^).

**Table 2:** Number of unique segregating sites in patients and controls.

Mitochondrial Region	Number of Unique segregating sites*		Number of Nonsynonymous changes		Number of Synonymous changes		Ratio of Nonsynonymous to Synonymous changes (P_N_/P_S_)		p value
	Patients	Controls	Patients	Controls	Patients	Controls	Patients	Controls	
**Coding Region**	243	147	74	41	169	106	0.44	0.39	0.5509
**Complex I**	114	68	36	12	78	56	0.46	0.21	**0.0053**
**Complex III**	27	22	12	8	15	14	0.80	0.57	0.2453
**Complex IV**	69	34	13	11	56	23	0.23	0.48	0.0225
**Complex V**	33	23	13	10	20	13	0.65	0.77	0.7334
**ND1**	17	9	7	3	10	6	0.70	0.50	1
**ND2**	15	13	4	4	11	9	0.36	0.44	1
**ND3**	5	3	1	0	4	3	0.25	0.00	–
**ND4**	22	10	3	1	19	9	0.16	0.11	1
**ND4L**	3	2	0	0	3	2	0.00	0.00	–
**ND5**	44	28	17	3	27	25	0.63	0.12	**0.0002**
**ND6**	8	3	4	1	4	2	1.00	0.50	0.69
**CYTB**	27	22	12	8	15	14	0.80	0.57	0.2453
**CO1**	37	16	5	5	32	11	0.16	0.45	0.0327
**CO2**	16	4	3	0	13	4	0.23	0.00	–
**CO3**	16	14	5	6	11	8	0.45	0.75	0.4138
**ATP6**	25	16	9	7	16	9	0.56	0.78	0.526
**ATP8**	8	7	4	3	4	4	1.00	0.75	1

* Unique segregating sites: Referred to the unique sites in patients or controls.

Next, among the variants in the coding region, the non-synonymous changes were segregated from the synonymous variants (Table 2). The ratio of the number of non-synonymous USS to the number of synonymous USS (P_N_/P_S_) did not vary significantly between patients and controls (0.44 vs 0.39, p = 0.5509). However, similar analysis on each of the four complexes (I, III, IV and V) separately within the coding sequence revealed that the nonsynonymous changes in Complex I was significantly higher in patients (p = 0.0053), highlighting the pathogenic involvement of the complexes in POAG (Table 2). Complex IV showed a marginally higher P_N_/P_S_ ratio in controls, primarily for the *COI* gene (p = 0.0327) (Table 2).

Subsequently, to delineate the complex in the coding region that might be associated with POAG, the distribution of USS for the nonsynonymous changes were assessed in each of the four mitochondrial complexes (I, III, IV and V), as detailed in the subsequent section.

#### Preponderance of non-synonymous changes in Complex I

**Figure 2:**
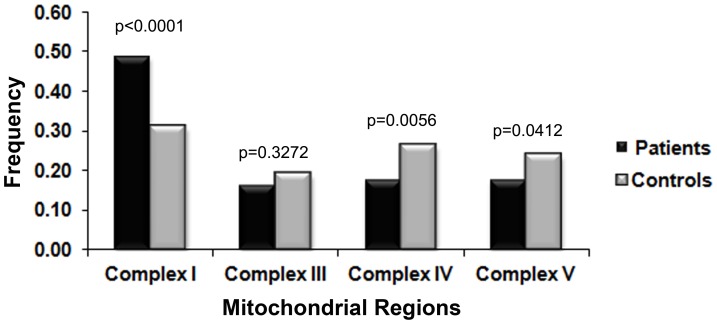
Distribution of non-synonymous unique segregating sites (USS) in the mtDNA complexes. The frequency of non-synonymous USS was significantly higher (p<0.0001) in patients compared to controls in the case of Complex I. Marginally higher segregating sites were observed in controls for Complex IV and Complex V.

In our dataset, the maximum number of non-synonymous USS were clustered in Complex I, in both patients (49%) and controls (31%) (Figure 2 & Table S2). The frequency of USS was significantly higher in patients compared to controls (0.49 vs 0.31, p<0.0001). The estimate of θ_w_ was 6.94 (±2) for patients and 2.48 (±0.94) for controls. This difference was extremely significant, with a p-value <1.49×10^−43^ (Table S4).

Not many significant observations were made in other complexes (Table S2 and Table S3). However, the mutation rate (θ_w_) was significantly higher in patients for all three of the other complexes as well (Table S4).

#### ND5 gene in complex I harbors maximum variations

**Figure 3:**
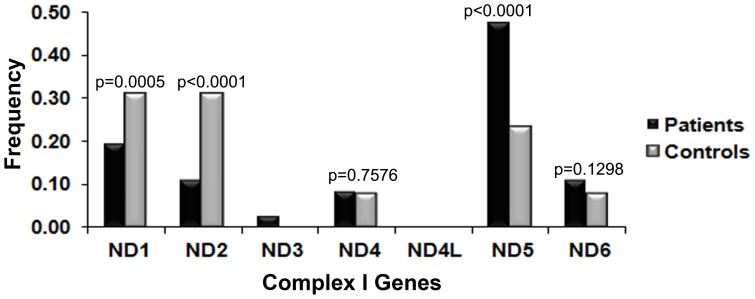
Distribution of non-synonymous unique segregating sites (USS) in Complex I genes. The frequency of non-synonymous USS was significantly higher (p<0.0001) in patients compared to controls in the case of the *ND5* gene. However, the frequency of USS was higher for controls in *ND1* and *ND2*.

When Complex I was further analyzed for the constituent seven genes (i.e. *ND1*, *ND2*, *ND3 ND4*, *ND4L*, *ND5* and *ND6*), we observed that the P_N_/P_S_ ratio of USS, was significantly higher in the patients for *ND5* (0.0002) (Table 2). This observation suggested that among the seven genes, variations in *ND5* are likely to play a role in POAG pathogenesis. Further dissection of the number of nonsynonymous USS in Complex I genes showed that the frequency of USS in *ND5* was significantly higher in patients than in controls (0.47 vs 0.23, p<0.0001) (Figure 3 & Table S5). The observation was further strengthened with the analysis of the mutational rate. The estimate of θ_w_ is 3.28 (±1.1) for patients and 0.62 (±0.38) for controls (Table S5). This difference was extremely significant with a p-value <4.42×10^−47^. This study deciphered the potential mitochondrial gene that might have a putative role in POAG pathogenesis for the first time.

Although *ND1* and *ND2* showed higher levels of variation in controls (Figure 3), the mutation rate (θ_w_) was higher in the patients for *ND1* (p = 3.3×10^−10^), and was not different for *ND2* (p = 0.118) (Table S5). Interestingly, all Complex I genes except *ND2* showed higher mutational rates in patients.

### Non Synonymous Changes Identified in POAG Patients

A total of 74 nonsynonymous mutations were identified in 101 POAG patients. The list of potentially damaging variants (as predicted by Polyphen-2) identified in patients is provided in Table 3. Among all of the mitochondrial genes, *ND5* was noted to harbor the maximum mutational load (24 patients harboring 17 variants). Among the variations identified in *ND5*, *in-silico* analysis with Polyphen 2 predicted four changes to be damaging viz. Thr331Ala, Thr412Ala, Leu(2)555Gln and Thr579Ala. Leu(2)555Gln (T14000A) was identified in 4 patients and was predicted to be highly pathogenic (Polyphen score: 0.00026), which points towards a potential role of the variant in POAG pathogenesis. Interestingly, all 4 of these patients developed the disease after 50 years of age and had a positive family history of glaucoma, but not observed to follow a specific pattern of inheritance was not observed.

**Table 3:** Non synonymous changes identified only in POAG patients.

Gene	Nucleotide Substitution	AA change*	Reported or Novel	Polyphen Prediction	Polyphen Score	Number of Patients
**ND1**	3979 A-C	Met225Leu(2)	Novel	Possibly damaging	0.803	1
**ND1**	3979 A-G	Met225Val	Novel	Possibly damaging	0.731	1
**ND2**	5178 C-A	Leu237Met	Reported	Possibly damaging	0.704	2
**ND2**	5504 A-C	Met345Ile	Novel	Possibly damaging	0.882	1
**COI**	6043 T-C	Leu47Pro	Novel	Probably damaging	0.964	1
**COI**	7182 C-T	Pro427Ser(1)	Novel	Probably damaging	0.988	8
**COII**	7750 C-G	Ile55Met	Novel	Probably damaging	0.273	2
**ATP8**	8387 G-A	Val8Met	Novel	Possibly damaging	0.719	1
**ATP6**	8572 G-A	Gly16Ser(2)	Reported	Probably damaging	0.82	1
**ATP6**	8945 T-C	Met140Thr	Reported	Possibly damaging	0.792	1
**ATP6**	9055 G-A	ALa177Thr	Unknown significance	Possibly damaging	0.835	5
**ATP6**	9119 T-C	Leu(2)198Pro	Novel (Sequence listing only in Gene bank)	Probably damaging	0.136	1
**COIII**	9903 T-C	Phe233Leu(2)	Reported in PhyloTree Build 14	Possibly damaging	0.136	1
**ND3**	10159 C-A	Ser34Tyr	Novel	Probably damaging	0.00018	1
**ND4**	11984 T-C	Tyr409His	Reported	Probably damaging	0.273	1
**ND5**	13327 A-G	Thr331Ala	Reported	Probably damaging	0.545	1
**ND5**	13570 A-G	Thr412Ala	Novel	Possibly damaging	0.883	1
**ND5**	14000 T-A	Leu(2)555Gln	Reported	Probably damaging	0.00026	4
**ND5**	14071 A-G	Thr579Ala	Reported	Possibly damaging	0.848	1
**CYTB**	15468 C-T	Thr241Met	Reported	Possibly damaging	0.821	1
**CYTB**	15675 C-T	Ser(1)310Phe	Novel	Possibly damaging	0.815	1

* AA: Amino Acid.

In addition to *ND5*, some major changes were identified in other genes. Next to *ND5*, the maximum number of variations was observed in *CYTB*. Twelve variations were identified in 18 patients but none in controls. Among these changes, Thr7Ile (T14766C) was found in 6 patients, predicted to be benign by Polyphen2. A notable observation was the presence of a single variant Ala177Thr (G9055A) in the *ATP6* gene in 5 patients. Another variant, Leu(2)198Pro (T9119C), predicted to be damaging by Polyphen was present in an early onset case (age at diagnosis –34 years). In addition, a few other damaging variants were also found sporadically in the other genes (Table 3).

### Analysis of Variants in the mtDNA RNA Region

**Figure 4:**
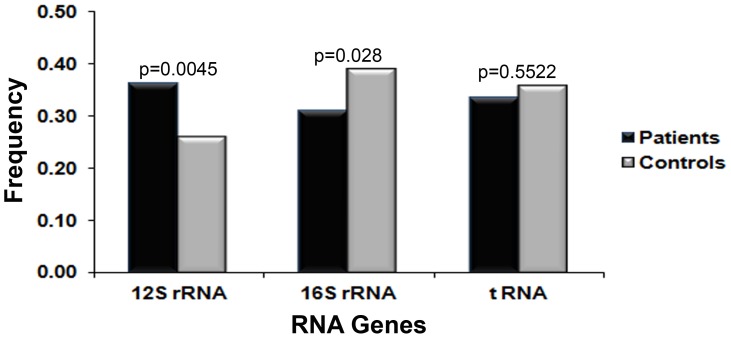
Distribution of unique segregating sites (USS) in the RNA genes. The frequency of USS was significantly higher (p = 0. 0045) in patients compared to controls in the *12S rRNA* gene.

The number of segregating sites in the *12S rRNA* gene was found to be significantly higher in patients compared to the controls (0.36 vs 0.23, p = 0.0004) (Figure 4 & Table S6). Interestingly, among the changes identified in *12S rRNA* (Table S7) in patients, T710C, C1375T, T1407C and A1438G are predicted to result in complete change of the RNA secondary structure. A substantial decrease in free energy was inferred for all 4 variants, which might affect the stability of 12S rRNA. The changes identified in 16S rRNA are listed in Table S7. In addition to these changes, one C insertion in between positions 3167 and 3168 was found in 2 patients (GL441 and GL598). None of the changes in 16S rRNA were predicted to alter RNA secondary structure.

No difference in the frequency of variants was observed in the tRNA genes between patients and controls. In our study, 13 variants in 10 tRNA genes were observed in 15 POAG patients (Table S8). A variant, T12285C, in tRNA-LeuCUN (Leu 2) was identified in 3 patients. The rest of the changes were identified in single patients. All identified changes (except one) were novel (Table S8).

### Analysis of Variants in the mtDNA Control Region

The D-loop region or the hypervariable region of the mitochondrial genome accounts for the maximum number of spontaneous variations. In our cohort, no significant observation was made with the variations identified in patients in the control region. However, two polymorphic positions in Hypervariable region I, C16261T and G16129A, were significantly associated with POAG with p values 6.4×10^−3^ (OR: 10.46) and 2.6×10^−2^ (OR: 2.618), respectively (Table 4). The entire list of Single Nucleotide Polymorphisms identified in patients and controls is provided in Table S9. Two insertion variations were found in the control region in patients, one C insertion in Hypervariable region I, in the Termination Associated Sequence between 16169 and 16170 (GL686), and another CC insertion in the noncoding region between 573 and 574 (GL441).

**Table 4:** SNPs associated with POAG.

Sl No	Nucleotide Position	Minor Allele	MAF in Patients	MAF in Controls	Major Allele	CHISQ	p value	OR
1	7441	T	0.01	0.09	C	6.012	1.4×10^−2^	0.107
2	13656	C	0.01	0.1	T	7.349	6.7×10^−3^	0.09184
3	16129	A	0.25	0.11	G	4.987	2.6×10^−2^	2.618
4	16145	A	0.09	0.01	G	4.325	3.7×10^−2^	6.9
5	16261	T	0.13	0.014	C	7.422	6.4×10^−3^	10.46

CHISQ: Chi-Square; MAF: Minor Allele Frequency; OR: Odds Ratio.

None of the haplogroup markers showed any association with POAG. Thus, it seems that in our population, it is not a particular haplogroup that portrays risk to POAG. An estimate of different haplogroups was made in the entire cohort (Table S10 and Table S11). The chi-squared test of homogeneity (p-value 0.205) suggests that the haplogroup frequency distribution is not different for the patient and control groups (Table 5). We observed that the number of distinct haplogroups in patients is more than that in controls, i.e. haplogroup C and D appear only in patients at a very low frequency −0.009 and 0.019, respectively (Table 5), and are unlikely to have any effect on the overall ‘genetic variation’. The ancient haplogroup L3 is also slightly greater in frequency in patients, but again owing to its low frequency would unlikely have an effect. The L3 haplogroup is considered to be the ancestor of both the M and N haplogroups, while N is thought to be the ancestor of R. L3, M, N and R are the major haplogroups that were observed in our dataset. The most divergent and wide-spread haplogroup of the subcontinent, haplogroup M, appears at a greater frequency (though not significant at 5%) in the controls. Thus, our data shows a higher proportion of the divergent haplogroup among the controls rather than the patients, supporting our claim of higher mutational rate among patients.

**Table 5:** Haplogroup frequencies in patients and controls *(Chi-squared test for homogeneity p-value is 0.205).*

Haplogroups	Frequency in Patients	Frequency in Controls
**L3**	0.089	0.069
**M**	0.56	0.69
**N**	0.019	0.028
**R**	0.18	0.07
**U**	0.069	0.13
**C**	0.009	–
**D**	0.019	–

## Discussion

In the present study we evaluated 101 POAG patients and 71 controls to investigate the possible involvement of mitochondrial genomic variants in POAG pathogenesis. It is known that mitochondrial DNA is more prone to mutation than genomic DNA. It is evident from the observation that the mitochondrial sequence between two individuals from any worldwide population differs on an average of 30bp substitutions [51]. For our study we primarily focused on the regions where the frequency of changes is higher in patients. This strategy was undertaken since we used mitochondria from the peripheral blood of the patients and controls, which would not only reveal changes associated with POAG, but also those related to other pathogeneses. In this context, it is worthwhile to mention that the ideal location for collection of mitochondria would be the site of pathogenesis, i.e the trabecular meshwork of the eye not normally accessible for the purpose of research. Such an endeavor would be more challenging with the need for a greater number of tissue samples. Numerous systemic and common disorders are also known to increase the risk of glaucoma [52]–[53]. However, any systemic difference between patients and controls observed in the analysis of blood samples is only likely to be pronounced when analyzed from the appropriate tissue.

After suitably adjusting for the sample size difference, the number of unique segregating sites in patients was found to be greater than that of controls; indicating that the patient pool acquired mutations at novel sites. The difference, however, was more pronounced when we compared only the coding sequence between patients and controls. The number of segregating sites was considerably higher in patients than in controls.

In both patients and controls, the ratio of transition to transversion (T_N_/T_V_) was higher in the coding region compared to the control region. A lower T_N_/T_V_ ratio, observed in the coding region of patients compared to controls, indicated the preponderance of transversion in patients. As transversions alter the chemical structure of the DNA drastically, the consequence of the change is more lethal than that of transitions. No selection pressure acts on mutations in the control region, so the T_N_/T_V_ ratio is comparable for patients and controls for this region. It was also observed that the clustering of nonsynonymous variants in the coding region of mtDNA was significantly higher in the *ND5* gene of Complex I in patients than in controls. In addition, the frequency of changes in *12S rRNA* was also significantly higher in patients than in controls. Although the number of segregating sites was significantly higher in controls in *ND1* and *ND2* of Complex I, the mutation rate was significantly higher in patients for all Complex I genes, except *ND2* (p = 0.188).

Mitochondrial Complex I is the entry port of the respiratory chain. It is an 850 kDa supramolecular complex composed of more than 40 polypeptides and contains a flavin mononucleotide (FMN) molecule and eight iron-sulphur clusters as redox active centers, embedded in the peripheral arm. Seven polypeptides of Complex I are encoded by mitochondrial genes (*ND-1, -2, -3, -4, -4L, -5,* and *-6*). Complex I catalyses the transfer of electrons from NADH via FMN to the metal redox pathway, and subsequently to ubiquinone (UQ), located in the membrane embedded part of the complex [54]. Thus, Complex I produces significant levels of ROS by molecular oxidation of O2, thereby accounting for most of the constitutive ROS generated by the mitochondrial respiratory chain [55]–[57]. The rate of ROS production is increased by electron transfer inhibition with rotenone (ROT), a hydrophobic pesticide [58]. Depending upon the physiological conditions or specific pathological modifications of Complex I, each of the redox active centers contributes to the ROS generating activity. However, the exact function of each ND subunit is not completely understood. Biochemical studies to date suggest the involvement of ND5 and ND6 in the proton pumping activity [59] as well as binding of ubiquinone [60]–[61].

Over the years, variants in Complex I have been linked to several mitochondrial and neurological diseases. A similar study of mitochondrial variations in POAG patients also reported clustering of mitochondrial variants in Complex I in a Saudi Arabian POAG cohort [32]. Similar observations have also been made in other optic neuropathies like LHON [62]–[63], and mitochondrial diseases viz. MERRF [64] and MELAS [65], where most of the mutations to date have been found in different Complex I genes. Defects in mitochondrial Complex I are known to be involved in increased production of ROS and are linked to several degenerative disorders [66]. Complex I deficiencies are also reported in devastating neurological disorders like Parkinson’s disease [67]–[68], Huntington’s disease [69] and Wilson’s disease [70].

Interestingly, a higher level of endogenous ROS has been observed in glaucomatous TM cells, and these cells were also found to be more sensitive to inhibition of Complex I activity by Rotenone (ROT) compared to normal tissue. On the other hand, inhibition of Complexes II and III had little effect on TM cells [33]. Inhibition with ROT resulted in further increase in ROS production, release of cytochrome C, and decrease in ATP level and mitochondrial membrane potential (ΔΨm) in glaucomatous TM cells, leading to apoptosis. This effect was found to be reversed with the use of antioxidants [33]. In the absence of any proofreading mechanism for the mitochondrial genome, there is a higher chance of mtDNA damage due to an accumulation of ROS in the mitochondria. The cycle of mtDNA damage and ROS production will hasten up in an already diseased cell; thus aggravating the disease pathogenesis. Such a phenomenon of mitochondrial dysfunction and reduced Complex I activity has been observed in other neurological disorders like Parkinson’s disease [71]. Reduced Complex I activity in Parkinson’s disease experimental animals intoxicated with Complex I inhibitors reproduces the clinical symptoms of Parkinson’s disease in humans [71]. Thus, our observation is likely to be of pathogenic importance even in the case of POAG, and might be associated with TM cell degeneration.

Although Complex I mutations have been identified in multiple instances, this is the first study to identify *ND5*, the largest subunit of Complex I, as the region harboring maximum variations in POAG patients. In Complex I, mutation in *ND5* has been consistently found in several disorders like LHON, MERRF, MELAS [72]. A recent study in *E. Coli* has shown that ND5 is involved in the proton pumping mechanism [73]. Thus, variation in *ND5* gene is expected to perturb the equilibrium of the respiratory chain, leading to inhibition of the overall activity of Complex I. Mutations in ND5 have also been associated with oxidative phosphorylation disorders [74]. Thus, downstream functional assays to determine the pathogenicity of the identified variants may help in better understanding of the role of *ND5* in POAG pathogenesis. On the other hand, it is tempting to speculate that a correlation exists between the significantly lower number of segregating sites for *ND1* and *ND2* in patients and an underlying protective mechanism for glaucoma. However, this cannot be substantiated due to limited knowledge regarding the functionality of the individual subunits of Complex I. Unfortunately, the limited number of studies done on the entire mtDNA in POAG did not provide the opportunity to compare our data with observations made in other cohorts. This observation needs to be vindicated in the future with further functional and genetic studies.

We also observed that POAG patients harbor a higher number of variations in the *12S rRNA* gene. However, no studies report the association of this gene with any eye disorder. The mitochondrial *12S rRNA* gene encodes the RNA of the small ribosomal subunit, which is remarkably similar in secondary structure to prokaryotic small subunit (16S-like) RNA. It helps assemble the amino acids into the functioning proteins that carry out oxidative phosphorylation. Defects in *12S rRNA* have been associated with Parkinson’s disease [75] and carcinoma [76]. However, defects in *12S rRNA* have primarily been associated with aminoglycoside-induced and non-syndromic hearing loss in multiple studies [77]–[79]. Aminoglycosides bind to 12S rRNA, causing mistranslation or premature termination of protein synthesis [80]–[81]. Individuals harboring *12S rRNA* variants are susceptible to aminoglycoside-induced hearing loss. Thus it would be interesting to examine whether the exposure of aminoglycosides, used in certain eye drops, can exacerbate the disease condition in POAG patients harboring variations in *12S rRNA* in a similar manner to that of hearing impairment. In this context it is worthwhile to mention that deposition of cochlin, a protein implicated in non-syndromic hearing loss, was found in glaucomatous TM cells [82]. No significant involvement was found for 16S rRNA.

Mitochondrial tRNAs play an important role in mtDNA translation because nuclear tRNAs are not transported into mitochondria in humans [83]–[84]. The mutations in *mt-tRNAs* can change the secondary structure and alter the tertiary structure, thus finally affecting the translation of mtDNA encoded genes. Many pathogenic mutations in *mt-tRNA* genes have been reported to be associated with human diseases, especially in the stem regions [85], but the molecular bases for the proposed associations have not been extensively investigated [85]–[88]. Over the last few years, an increasing number of human neurodegenerative disorders have been found to be correlated with point mutations in *mt-tRNA*. Both the number of mutations (>70) and widely variable phenotypes (e.g. cardiopathies, myopathies, encephalopathies, diabetes, deafness) render the understanding of the genotype to phenotype relationships very complex [89]. From this perspective, studies on the structural changes occurring due to respective mutation in POAG patients are worth pursuing.

Our analysis revealed that the number of variants in coding regions is significantly different between the patients and controls, whereas no such bias was observed in the control regions. Thus, it is interesting to note that defects in mtDNA in POAG patients are more pronounced in the coding region, which might finally result in the disruption of the electron transport chain, leading to mitochondrial dysfunction.

There are numerous reports associating mtDNA haplogroups to various diseases including optic neuropathies [90]. In our study, a possible association between mtDNA haplogroups and POAG was investigated, but no such evidence was detected. Such a lack of association of mitochondrial haplogroups has also been observed in a study performed on a Caucasian population in Europe [91]. A similar observation was made in a study from Saudi Arabia [92]. However, a recent study by the same group showed a higher preponderance of haplogroup L in Saudi Arabian POAG patients [93]. They attributed this observation to unknown racial risk factors for the disease or unexpected population substructure in Saudi-Arabia. Mitochondrial DNA sequences belonging to macro haplogroup L has an African origin. It is well documented that individuals of African descent have a higher preponderance of glaucoma than Eurasians [94]. Our study population belongs to the Indo-European ethnic group, who do not have any African ancestry [43]. In India most of the population belongs to the M macro haplogroup [95], also observed in our dataset, which is primarily of Eurasian ancestry. Our observations are thus consistent with the published literature on similar population groups.

One of the limitations of this study is the use of the Sanger sequencing method for detection of mtDNA variations. Although the entire mtDNA had at least 2X coverage, this method fails to identify low levels of hetreoplasmy in the DNA [96]. Thus our data does not contain the changes present in very low frequency but only those which are more abundant.

In conclusion, we observed that the mutation rate of mtDNA is significantly higher in patients compared to the controls. Complex I was found to harbor the majority of the observed variants, and *ND5* seems to be the predominant player in POAG pathogenesis. Mutations in *ND5* are expected to inhibit Complex I activity, which in turn might lead to generation of oxidative stress and favor a glaucomatous condition.

## Supporting Information

Table S1 **Frequency of segregating sites in mtDNA.**
(DOCX)

Table S2 **Frequency of non-synonymous USS in mitochondrial complexes in patients and controls.**
(DOCX)

Table S3 **Frequency of non-synonymous USS in Complex IV and Complex V genes.**
(DOCX)

Table S4 **Watterson’s θ for non-synonymous USS in mitochondrial complexes in patients and controls.**
(DOCX)

Table S5 **Frequency of non-synonymous USS in Complex I genes.**
(DOCX)

Table S6 **Frequency of USS in RNA genes in patients and controls.**
(DOCX)

Table S7 **Variations identified in rRNA genes in patients.**
(DOCX)

Table S8 **Variations identified in tRNA genes in patients.**
(DOCX)

Table S9 **SNPs identified in mitochondrial genome.**
(DOCX)

Table S10 **List of haplogroups of individual patients.**
(DOCX)

Table S11 **List of haplogroups of individual controls.**
(DOCX)
