# Supplementary material for: Mitochondrial Genome Analysis of Primary Open Angle Glaucoma Patients
Source: PLoS One. 2013 Aug 5;8(8):e70760. doi: 10.1371/journal.pone.0070760 (PMC3733777; doi:10.1371/journal.pone.0070760)
Supplement: Table S1 — Frequency of segregating sites in mtDNA. (DOCX) [file pone.0070760.s001.docx]

**Table S1: Frequency of segregating sites in mtDNA**

| **Mitochondrial regions** | **Segregating sites** | | **p value** |
| --- | --- | --- | --- |
|  | **Frequency in Patients (n)** | **Frequency in Controls (n)** |  |
| **Coding region** | 0.69 (243) | 0.62 (147) | **0.0347** |
| **RNA genes** | 0.11 (39) | 0.13 (31) | 0.3998 |
| **Control region** | 0.20 (69) | 0.25 (58) | 0.0982 |

Estimate of Watterson’s θ (± SD) is provided in the text
