# Supplementary material for: Mitochondrial Genome Analysis of Primary Open Angle Glaucoma Patients
Source: PLoS One. 2013 Aug 5;8(8):e70760. doi: 10.1371/journal.pone.0070760 (PMC3733777; doi:10.1371/journal.pone.0070760)
Supplement: Table S2 — Frequency of non-synonymous USS in mitochondrial complexes in patients and controls. (DOCX) [file pone.0070760.s002.docx]

**Table S2: Frequency of non-synonymous USS in mitochondrial complexes in patients and controls**

| **Mitochondrial regions** | **Segregating sites** | | **p value** |
| --- | --- | --- | --- |
|  | **Frequency in Patients (n)** | **Frequency in Controls (n)** |  |
| **Complex I** | 0.49 (36) | 0.31 (12) | **<0.0001** |
| **Complex III** | 0.16 (12) | 0.19 (8) | 0.3272 |
| **Complex IV** | 0.18 (13) | 0.26 (11) | 0.0056 |
| **Complex V** | 0.18 (13) | 0.24 (10) | 0.0412 |

*USS: Unique Segregating Sites
