# Supplementary material for: Mitochondrial Genome Analysis of Primary Open Angle Glaucoma Patients
Source: PLoS One. 2013 Aug 5;8(8):e70760. doi: 10.1371/journal.pone.0070760 (PMC3733777; doi:10.1371/journal.pone.0070760)
Supplement: Table S3 — Frequency of non-synonymous USS in Complex IV and Complex V genes. (DOCX) [file pone.0070760.s003.docx]

**Table S3: Frequency of non-synonymous USS in Complex IV and Complex V genes**

| **Mitochondrial regions** | **Genes** | **Segregating sites** | | **p value** |
| --- | --- | --- | --- | --- |
|  |  | **Frequency in Patients (n)** | **Frequency in Controls (n)** |  |
| **Complex IV** | **COI** | 0.38 (5) | 0.45 (5) | 0.0626 |
|  | **COII** | 0.23 (3) | 0 | - |
|  | **COIII** | 0.38 (5) | 0.55 (6) | <0.0001 |
| **Complex V** | **ATP6** | 0.69 (9) | 0.70 (7) | 0.8265 |
|  | **ATP8** | 0.31 (4) | 0.30 (3) | 0.8265 |

*USS: Unique Segregating Sites
