# Supplementary material for: Mitochondrial Genome Analysis of Primary Open Angle Glaucoma Patients
Source: PLoS One. 2013 Aug 5;8(8):e70760. doi: 10.1371/journal.pone.0070760 (PMC3733777; doi:10.1371/journal.pone.0070760)
Supplement: Table S4 — Watterson’s θ for non-synonymous USS in mitochondrial complexes in patients and controls. (DOCX) [file pone.0070760.s004.docx]

**Table S4: Watterson’s θ for non-synonymous USS in mitochondrial complexes in patients and controls**

| **Mitochondrial regions** | **Watterson’s θ (±SD)** | | **p value** |
| --- | --- | --- | --- |
|  | **Estimate in Patients (±SD)** | **Estimate in Controls (±SD)** |  |
| **Complex I** | 6.94 (2) | 2.48 (0.94) | **1.49 X 10^-43^** |
| **Complex III** | 2.31(0.85) | 1.66(0.71) | 9.28 X 10^-8^ |
| **Complex IV** | 2.51(0.9) | 2.28(0.88) | 0.042 |
| **Complex V** | 2.51(0.9) | 2.07(0.82) | 0.0005 |

*USS: Unique Segregating Sites
