# Supplementary material for: Mitochondrial Genome Analysis of Primary Open Angle Glaucoma Patients
Source: PLoS One. 2013 Aug 5;8(8):e70760. doi: 10.1371/journal.pone.0070760 (PMC3733777; doi:10.1371/journal.pone.0070760)
Supplement: Table S5 — Frequency of non-synonymous USS in Complex I genes. (DOCX) [file pone.0070760.s005.docx]

**Table S5: Frequency of non-synonymous USS in Complex I genes**

| **Complex I genes** | **Segregating sites** | | **p value** | **Watterson’s θ (±SD)** | | **p value** |
| --- | --- | --- | --- | --- | --- | --- |
|  | **Frequency in Patients (n)** | **Frequency in Controls (n)** |  | **Estimate in Patients (±SD)** | **Estimate in Controls (±SD)** |  |
| **ND1** | 0.19 (7) | 0.31 (4) | 0.0005 | 1.35 (0.59) | 0.83 (0.45) | 3.3 X 10^-10^ |
| **ND2** | 0.11 (4) | 0.31 (4) | <0.0001 | 0.77(0.42) | 0.83 (0.45) | 0.188 |
| **ND3** | 0.03 (1) | 0 | - | 0.19(0.19) | 0 | - |
| **ND4** | 0.08 (3) | 0.08 (1) | 0.7576 | 0.58(0.35) | 0.21 (0.21) | 2.2 X 10^-15^ |
| **ND4L** | 0 | 0 | - | 0 | 0 | - |
| **ND5** | 0.47 (17) | 0.23 (3) | **<0.0001** | 3.27 (1.1) | 0.62 (0.38) | **5.6 X 10^-47^** |
| **ND6** | 0.11 (4) | 0.08 (1) | 0.1298 | 0.77 (0.41) | 0.21 (0.21) | 7.2 X 10^-23^ |

*USS: Unique Segregating Sites
