# Supplementary material for: Mitochondrial Genome Analysis of Primary Open Angle Glaucoma Patients
Source: PLoS One. 2013 Aug 5;8(8):e70760. doi: 10.1371/journal.pone.0070760 (PMC3733777; doi:10.1371/journal.pone.0070760)
Supplement: Table S6 — Frequency of USS in RNA genes in patients and controls. (DOCX) [file pone.0070760.s006.docx]

**Table S6: Frequency of USS in RNA genes in patients and controls**

| **RNA genes** | **Segregating sites** | | **p value** |
| --- | --- | --- | --- |
|  | **Frequency in Patients (n)** | **Frequency in Controls (n)** |  |
| **12S rRNA** | 0.36 (14) | 0.26 (8) | **0.0045** |
| **16S rRNA** | 0.31 (12) | 0.39 (12) | 0.028 |
| **tRNA** | 0.33 (13) | 0.35 (11) | 0.5522 |

*USS: Unique Segregating Sites
