# Supplementary material for: Mitochondrial Genome Analysis of Primary Open Angle Glaucoma Patients
Source: PLoS One. 2013 Aug 5;8(8):e70760. doi: 10.1371/journal.pone.0070760 (PMC3733777; doi:10.1371/journal.pone.0070760)
Supplement: Table S7 — Variations identified in rRNA genes in patients. (DOCX) [file pone.0070760.s007.docx]

**Table S7: Variations identified in rRNA genes in patients**

| **RNA genes** | **Variation** | **Nucleotide Position (bp)** | **Reported or novel** | **No of Patients** | **Change in free energy due to structural change** |
| --- | --- | --- | --- | --- | --- |
| **12S rRNA** | **C702T** | 55 | Novel | 1 | No change |
|  | **C710T** | 63 | Novel | 1 | -7.77 |
|  | **C808T** | 161 | Novel | 1 | No change |
|  | **T953C** | 306 | Novel | 1 | No change |
|  | **T980C** | 333 | Novel | 1 | No change |
|  | **A1025G** | 378 | Novel | 1 | No change |
|  | **C1088A** | 441 | Novel | 1 | No change |
|  | **T1284C** | 637 | Novel | 1 | No change |
|  | **C1375T** | 728 | Novel | 1 | -8.92 |
|  | **T1407C** | 760 | Novel | 2 | -4.73 |
|  | **A1438G** | 791 | Associated with Schizophrenia | 1 | -8.26 |
|  | **G1442A** | 795 | Novel | 2 | No change |
|  | **C1531T** | 884 | Novel | 2 | No change |
|  | **A1585G** | 938 | Novel | 1 | No change |
|  |  |  |  |  |  |
|  |  |  |  |  |  |
| **16S rRNA** | **A1692G** | 22 | Novel | 2 | No change |
|  | **T1752A** | 82 | Novel | 1 | No change |
|  | **A1978C** | 308 | Novel | 1 | No change |
|  | **T2404C** | 734 | Novel | 1 | No change |
|  | **T2442A** | 772 | Novel | 1 | No change |
|  | **A2581G** | 911 | Novel | 1 | No change |
|  | **C2708A** | 1038 | Novel | 1 | No change |
|  | **T3083C** | 1413 | Novel | 1 | No change |
|  | **C3110T** | 1439 | Novel | 1 | No change |
|  | **C1445T** | 1445 | Novel | 1 | No change |
|  | **G3163A** | 1492 | Novel | 1 | No change |
