# Supplementary material for: Mitochondrial Genome Analysis of Primary Open Angle Glaucoma Patients
Source: PLoS One. 2013 Aug 5;8(8):e70760. doi: 10.1371/journal.pone.0070760 (PMC3733777; doi:10.1371/journal.pone.0070760)
Supplement: Table S8 — Variations identified in tRNA genes in patients. (DOCX) [file pone.0070760.s008.docx]

**Table S8: Variations identified in tRNA genes in patients**

| **Variation** | **Location** | **Reported or novel** | **No of Patients** |
| --- | --- | --- | --- |
| **G622A** | tRNA-Phe, Nucleotide 45 | EXIT & Deafness | 1 |
| **A3267T** | tRNA-LeuUUR, Nucleotide 36 in anticodon | Novel | 1 |
| **T4386C** | tRNA-Glu, Nucleotide 15 in DHU Loop | Novel | 1 |
| **C5601T** | tRNA-Ala, Nucleotide 59 in T stem | Novel | 1 |
| **C5784G** | tRNA-Cys, Nucleotide 49 in T stem , L strand replication origin (5721-5798) | Novel | 1 |
| **T5814C** | tRNA-Cys, Nucleotide 13 in DHU stem | Mitochondrial Encephalopathy | 1 |
| **A5823G** | tRNA-Cys, Nucleotide 4 in acceptor stem | Novel | 1 |
| **T10463C** | tRNA-Arg, Nucleotide 67 in the acceptor stem | Novel | 1 |
| **A12172G** | tRNA-His, nucleotide 38 in the anticodon loop | Novel | 1 |
| **A12265C** | tRNA-SerAGY(Ser 2), Nucleotide 73 | Novel | 1 |
| **A12280G** | tRNA-LeuCUN(Leu 2), Nucleotide 15 in the DHU loop | Novel | 1 |
| **T12285C** | tRNA-LeuCUN(Leu 2), Nucleotide 20 in the DHU loop | Novel | 3 |
| **T15892C** | tRNA-Thr, Nucleotide 5 in acceptor stem | Novel | 1 |
