# Supplementary material for: Mitochondrial Genome Analysis of Primary Open Angle Glaucoma Patients
Source: PLoS One. 2013 Aug 5;8(8):e70760. doi: 10.1371/journal.pone.0070760 (PMC3733777; doi:10.1371/journal.pone.0070760)
Supplement: Table S9 — SNPs identified in mitochondrial genome. (DOCX) [file pone.0070760.s009.docx]

**Table S9: SNPs identified in mitochondrial genome**

| **Sl No** | **Nucleotide Position** | **Minor Allele** | **MAF in Patients** | **MAF in Controls** | **Major Allele** | **CHISQ** | **p value** | **OR** |
| --- | --- | --- | --- | --- | --- | --- | --- | --- |
| 1 | 143 | A | 0.06 | 0.01 | G | 2.193 | 0.139 | 4.421 |
| 2 | 146 | C | 0.18 | 0.18 | T | 0.006722 | 0.935 | 0.9676 |
| 3 | 151 | T | 0.03 | 0.03 | C | 0.003476 | 0.953 | 1.056 |
| 4 | 152 | C | 0.35 | 0.25 | T | 1.692 | 0.193 | 1.561 |
| 5 | 189 | G | 0.04 | 0.01 | A | 0.962 | 0.327 | 2.887 |
| 6 | 198 | T | 0.04 | 0.01 | C | 0.962 | 0.327 | 2.887 |
| 7 | 199 | C | 0.06 | 0.08 | T | 0.4048 | 0.525 | 0.6842 |
| 8 | 200 | G | 0.04 | 0.01 | A | 0.962 | 0.327 | 2.887 |
| 9 | 204 | C | 0.09 | 0.10 | T | 0.04443 | 0.833 | 0.8944 |
| 10 | 207 | A | 0.03 | 0.07 | G | 1.559 | 0.212 | 0.4041 |
| 11 | 209 | G | 0.01 | 0.01 | T | 0.06349 | 0.801 | 0.7 |
| 12 | 210 | G | 0.01 | 0.01 | A | 0.06349 | 0.801 | 0.7 |
| 13 | 215 | G | 0.03 | 0.01 | A | 0.4477 | 0.503 | 2.143 |
| 14 | 228 | A | 0.02 | 0.01 | G | 0.07953 | 0.778 | 1.414 |
| 15 | 234 | G | 0.03 | 0.07 | A | 1.559 | 0.212 | 0.4041 |
| 16 | 235 | G | 0.02 | 0.03 | A | 0.1285 | 0.720 | 0.697 |
| 17 | 246 | C | 0.03 | 0.04 | T | 0.1951 | 0.659 | 0.6939 |
| 18 | 250 | C | 0.01 | 0.01 | T | 0.06349 | 0.801 | 0.7 |
| 19 | 324 | G | 0.01 | 0.03 | C | 0.8119 | 0.368 | 0.345 |
| 20 | 366 | A | 0.01 | 0.04 | G | 1.921 | 0.166 | 0.2267 |
| 21 | 461 | T | 0.01 | 0.04 | C | 1.679 | 0.195 | 0.2462 |
| 22 | 482 | C | 0.08 | 0.13 | T | 1.215 | 0.270 | 0.5596 |
| 23 | 489 | T | 0.31 | 0.29 | C | 0.07624 | 0.783 | 1.102 |
| 24 | 575 | T | 0.01 | 0.01 | C | 0.0369 | 0.848 | 0.7614 |
| 25 | 709 | A | 0.16 | 0.18 | G | 0.1025 | 0.749 | 0.8711 |
| 26 | 1243 | C | 0.03 | 0.02 | T | 0.3615 | 0.548 | 1.99 |
| 27 | 1406 | C | 0.01 | 0.02 | T | 0.093 | 0.760 | 0.65 |
| 28 | 1462 | A | 0.01 | 0.02 | G | 0.093 | 0.760 | 0.65 |
| 29 | 1598 | A | 0.04 | 0.04 | G | 0.007144 | 0.933 | 0.9362 |
| 30 | 1719 | A | 0.02 | 0.03 | G | 0.1274 | 0.721 | 0.6979 |
| 31 | 1780 | C | 0.01 | 0.03 | T | 0.8096 | 0.368 | 0.3454 |
| 32 | 1811 | G | 0.11 | 0.14 | A | 0.3934 | 0.531 | 0.746 |
| 33 | 1888 | A | 0.14 | 0.16 | G | 0.08727 | 0.768 | 0.8788 |
| 34 | 2361 | A | 0.02 | 0.03 | G | 0.1495 | 0.699 | 0.6774 |
| 35 | 2706 | A | 0.07 | 0.09 | G | 0.1793 | 0.672 | 0.7822 |
| 36 | 3010 | A | 0.04 | 0.03 | G | 0.2147 | 0.643 | 1.5 |
| 37 | 3308 | C | 0.01 | 0.01 | T | 0.0464 | 0.829 | 0.7368 |
| 38 | 3486 | T | 0.01 | 0.03 | C | 0.7291 | 0.393 | 0.3632 |
| 39 | 3505 | G | 0.01 | 0.01 | A | 0.0464 | 0.829 | 0.7368 |
| 40 | 3537 | G | 0.02 | 0.07 | A | 2.499 | 0.114 | 0.2809 |
| 41 | 3591 | A | 0.01 | 0.01 | G | 0.0464 | 0.829 | 0.7368 |
| 42 | 3741 | T | 0.01 | 0.01 | C | 0.06349 | 0.801 | 0.7 |
| 43 | 3744 | G | 0.04 | 0.01 | A | 0.962 | 0.327 | 2.887 |
| 44 | 3780 | T | 0.01 | 0.04 | C | 1.921 | 0.166 | 0.2267 |
| 45 | 3915 | A | 0.01 | 0.01 | G | 0.06349 | 0.801 | 0.7 |
| 46 | 3921 | T | 0.06 | 0.04 | C | 0.2474 | 0.619 | 1.432 |
| 47 | 3970 | T | 0.01 | 0.01 | C | 0.06349 | 0.801 | 0.7 |
| 48 | 4099 | T | 0.01 | 0.01 | C | 0.06349 | 0.801 | 0.7 |
| 49 | 4216 | C | 0.01 | 0.01 | T | 0.06349 | 0.801 | 0.7 |
| 50 | 4394 | T | 0.02 | 0.02 | C | 0.02976 | 0.863 | 1.237 |
| 51 | 4454 | C | 0.02 | 0.02 | T | 0.02976 | 0.863 | 1.237 |
| 52 | 4491 | A | 0.02 | 0.02 | G | 0.02976 | 0.863 | 1.237 |
| 53 | 4580 | A | 0.06 | 0.11 | G | 1.482 | 0.223 | 0.4977 |
| 54 | 4646 | C | 0.01 | 0.02 | T | 0.1211 | 0.728 | 0.6122 |
| 55 | 4703 | C | 0.06 | 0.07 | T | 0.0159 | 0.900 | 0.9194 |
| 56 | 5186 | T | 0.03 | 0.06 | A | 0.744 | 0.388 | 0.5156 |
| 57 | 5252 | A | 0.02 | 0.03 | G | 0.1243 | 0.724 | 0.701 |
| 58 | 5301 | G | 0.01 | 0.04 | A | 1.904 | 0.168 | 0.2279 |
| 59 | 5319 | G | 0.03 | 0.03 | A | 0.004283 | 0.948 | 1.062 |
| 60 | 5360 | T | 0.01 | 0.01 | C | 0.0614 | 0.804 | 0.7041 |
| 61 | 5423 | G | 0.01 | 0.01 | A | 0.0614 | 0.804 | 0.7041 |
| 62 | 5460 | A | 0.02 | 0.03 | G | 0.1317 | 0.717 | 0.6939 |
| 63 | 5471 | A | 0.01 | 0.01 | G | 0.0614 | 0.804 | 0.7041 |
| 64 | 5558 | G | 0.01 | 0.04 | A | 1.904 | 0.168 | 0.2279 |
| 65 | 6190 | A | 0.04 | 0.03 | G | 0.01487 | 0.903 | 1.12 |
| 66 | 6253 | C | 0.03 | 0.01 | T | 0.4265 | 0.514 | 2.106 |
| 67 | 6340 | T | 0.02 | 0.01 | C | 0.07152 | 0.789 | 1.389 |
| 68 | 6392 | C | 0.01 | 0.01 | T | 0.0701 | 0.791 | 0.6875 |
| 69 | 6806 | G | 0.01 | 0.01 | A | 0.06835 | 0.794 | 0.6907 |
| 70 | 6962 | A | 0.03 | 0.03 | G | 0.01081 | 0.917 | 1.101 |
| 71 | 7196 | A | 0.02 | 0.01 | C | 0.07624 | 0.783 | 1.404 |
| 72 | 7441 | T | 0.01 | 0.09 | C | 6.012 | 0.014 | 0.107 |
| 73 | 7521 | A | 0.01 | 0.01 | G | 0.0464 | 0.829 | 0.7368 |
| 74 | 7581 | C | 0.01 | 0.01 | T | 0.06619 | 0.797 | 0.6947 |
| 75 | 7598 | A | 0.01 | 0.03 | G | 0.8249 | 0.364 | 0.3421 |
| 76 | 7610 | A | 0.04 | 0.04 | C | 0.009283 | 0.923 | 0.9275 |
| 77 | 7669 | A | 0.02 | 0.03 | C | 0.134 | 0.714 | 0.6915 |
| 78 | 7754 | A | 0.01 | 0.01 | G | 0.06619 | 0.797 | 0.6947 |
| 79 | 7759 | C | 0.01 | 0.01 | T | 0.06619 | 0.797 | 0.6947 |
| 80 | 7961 | C | 0.03 | 0.01 | T | 0.4683 | 0.494 | 2.179 |
| 81 | 8005 | C | 0.01 | 0.01 | T | 0.05783 | 0.810 | 0.7113 |
| 82 | 8023 | C | 0.02 | 0.01 | T | 0.08727 | 0.768 | 1.438 |
| 83 | 8137 | T | 0.01 | 0.01 | C | 0.05783 | 0.810 | 0.7113 |
| 84 | 8251 | A | 0.04 | 0.09 | G | 1.47 | 0.225 | 0.4539 |
| 85 | 8396 | G | 0.01 | 0.01 | A | 0.05783 | 0.810 | 0.7113 |
| 86 | 8502 | G | 0.01 | 0.03 | A | 0.7855 | 0.376 | 0.3505 |
| 87 | 8584 | A | 0.03 | 0.06 | G | 0.7929 | 0.373 | 0.5051 |
| 88 | 8594 | C | 0.05 | 0.03 | T | 0.4615 | 0.497 | 1.771 |
| 89 | 8676 | T | 0.03 | 0.01 | C | 0.4302 | 0.512 | 2.112 |
| 90 | 8684 | T | 0.01 | 0.01 | C | 0.06877 | 0.793 | 0.69 |
| 91 | 8701 | A | 0.35 | 0.32 | G | 0.1274 | 0.721 | 1.128 |
| 92 | 8887 | G | 0.01 | 0.01 | A | 0.06877 | 0.793 | 0.69 |
| 93 | 8994 | A | 0.01 | 0.01 | G | 0.06877 | 0.793 | 0.69 |
| 94 | 9053 | A | 0.01 | 0.03 | G | 0.8362 | 0.361 | 0.34 |
| 95 | 9064 | A | 0.04 | 0.04 | G | 0.01115 | 0.916 | 0.921 |
| 96 | 9094 | T | 0.02 | 0.06 | C | 1.703 | 0.192 | 0.3333 |
| 97 | 9098 | C | 0.01 | 0.01 | T | 0.06877 | 0.793 | 0.69 |
| 98 | 9139 | A | 0.01 | 0.03 | G | 0.8362 | 0.361 | 0.34 |
| 99 | 9317 | T | 0.01 | 0.01 | C | 0.05993 | 0.807 | 0.7071 |
| 100 | 9329 | A | 0.01 | 0.03 | G | 0.7952 | 0.373 | 0.3485 |
| 101 | 9389 | G | 0.01 | 0.01 | A | 0.05993 | 0.807 | 0.7071 |
| 102 | 9449 | T | 0.01 | 0.01 | C | 0.05993 | 0.807 | 0.7071 |
| 103 | 9540 | T | 0.38 | 0.30 | C | 1.303 | 0.254 | 1.459 |
| 104 | 9575 | A | 0.02 | 0.01 | G | 0.0843 | 0.772 | 1.429 |
| 105 | 9614 | G | 0.01 | 0.06 | A | 3.141 | 0.076 | 0.1692 |
| 106 | 9758 | C | 0.01 | 0.03 | T | 0.7952 | 0.373 | 0.3485 |
| 107 | 9767 | T | 0.03 | 0.01 | C | 0.4604 | 0.497 | 2.165 |
| 108 | 9773 | T | 0.03 | 0.01 | C | 0.4604 | 0.497 | 2.165 |
| 109 | 9804 | A | 0.01 | 0.01 | G | 0.05993 | 0.807 | 0.7071 |
| 110 | 9947 | A | 0.02 | 0.01 | G | 0.0843 | 0.772 | 1.429 |
| 111 | 9950 | C | 0.01 | 0.04 | T | 1.891 | 0.169 | 0.229 |
| 112 | 10142 | T | 0.01 | 0.01 | C | 0.04544 | 0.831 | 0.7391 |
| 113 | 10192 | T | 0.01 | 0.01 | C | 0.04544 | 0.831 | 0.7391 |
| 114 | 10238 | A | 0.05 | 0.01 | T | 1.6 | 0.206 | 3.71 |
| 115 | 10245 | C | 0.01 | 0.01 | T | 0.04544 | 0.831 | 0.7391 |
| 116 | 10310 | A | 0.02 | 0.03 | G | 0.1171 | 0.732 | 0.7083 |
| 117 | 10365 | A | 0.03 | 0.04 | G | 0.1398 | 0.709 | 0.7333 |
| 118 | 10398 | A | 0.37 | 0.30 | G | 0.8262 | 0.363 | 1.355 |
| 119 | 10400 | C | 0.34 | 0.29 | T | 0.5345 | 0.465 | 1.285 |
| 120 | 10556 | T | 0.03 | 0.01 | C | 0.5191 | 0.471 | 2.267 |
| 121 | 10589 | A | 0.01 | 0.03 | G | 0.7245 | 0.395 | 0.3641 |
| 122 | 10609 | C | 0.02 | 0.01 | T | 0.1072 | 0.743 | 1.495 |
| 123 | 10632 | C | 0.03 | 0.03 | T | 0.01418 | 0.905 | 1.117 |
| 124 | 10640 | C | 0.01 | 0.04 | T | 1.762 | 0.184 | 0.2391 |
| 125 | 10688 | A | 0.01 | 0.01 | G | 0.05783 | 0.810 | 0.7113 |
| 126 | 10754 | G | 0.04 | 0.03 | A | 0.1677 | 0.682 | 1.432 |
| 127 | 10774 | T | 0.01 | 0.01 | C | 0.0614 | 0.804 | 0.7041 |
| 128 | 10873 | T | 0.38 | 0.34 | C | 0.2844 | 0.594 | 1.194 |
| 129 | 10915 | T | 0.01 | 0.03 | C | 0.7786 | 0.378 | 0.352 |
| 130 | 11050 | C | 0.01 | 0.01 | T | 0.0614 | 0.804 | 0.7041 |
| 131 | 11075 | C | 0.02 | 0.01 | T | 0.08232 | 0.774 | 1.423 |
| 132 | 11083 | G | 0.01 | 0.04 | A | 1.904 | 0.168 | 0.2279 |
| 133 | 11084 | G | 0.01 | 0.01 | A | 0.0614 | 0.804 | 0.7041 |
| 134 | 11152 | C | 0.01 | 0.01 | T | 0.0614 | 0.804 | 0.7041 |
| 135 | 11251 | G | 0.01 | 0.01 | A | 0.05643 | 0.812 | 0.7143 |
| 136 | 11293 | G | 0.05 | 0.03 | A | 0.4969 | 0.481 | 1.809 |
| 137 | 11465 | C | 0.01 | 0.03 | T | 0.8096 | 0.368 | 0.3454 |
| 138 | 11467 | G | 0.10 | 0.16 | A | 1.242 | 0.265 | 0.596 |
| 139 | 11674 | T | 0.01 | 0.01 | C | 0.06294 | 0.802 | 0.701 |
| 140 | 11890 | G | 0.01 | 0.01 | A | 0.06294 | 0.802 | 0.701 |
| 141 | 11947 | G | 0.01 | 0.01 | A | 0.06667 | 0.796 | 0.6939 |
| 142 | 11969 | A | 0.01 | 0.01 | G | 0.06667 | 0.796 | 0.6939 |
| 143 | 12007 | A | 0.13 | 0.19 | G | 0.9575 | 0.328 | 0.6588 |
| 144 | 12308 | G | 0.11 | 0.15 | A | 0.7915 | 0.374 | 0.6667 |
| 145 | 12372 | A | 0.12 | 0.17 | G | 0.8751 | 0.350 | 0.6629 |
| 146 | 12406 | A | 0.03 | 0.01 | G | 0.4477 | 0.503 | 2.143 |
| 147 | 12414 | C | 0.01 | 0.01 | T | 0.06349 | 0.801 | 0.7 |
| 148 | 12477 | C | 0.08 | 0.06 | T | 0.336 | 0.562 | 1.441 |
| 149 | 12498 | C | 0.03 | 0.01 | T | 0.4477 | 0.503 | 2.143 |
| 150 | 12501 | A | 0.01 | 0.04 | G | 1.921 | 0.166 | 0.2267 |
| 151 | 12507 | G | 0.02 | 0.01 | A | 0.07953 | 0.778 | 1.414 |
| 152 | 12705 | C | 0.35 | 0.27 | T | 1.206 | 0.272 | 1.451 |
| 153 | 12810 | G | 0.01 | 0.04 | A | 1.921 | 0.166 | 0.2267 |
| 154 | 12882 | T | 0.01 | 0.01 | C | 0.06349 | 0.801 | 0.7 |
| 155 | 13135 | A | 0.05 | 0.03 | G | 0.4861 | 0.486 | 1.797 |
| 156 | 13145 | A | 0.01 | 0.01 | G | 0.06349 | 0.801 | 0.7 |
| 157 | 13174 | C | 0.01 | 0.01 | T | 0.06349 | 0.801 | 0.7 |
| 158 | 13194 | A | 0.04 | 0.07 | G | 0.7986 | 0.372 | 0.5443 |
| 159 | 13263 | G | 0.03 | 0.01 | A | 0.4477 | 0.503 | 2.143 |
| 160 | 13368 | A | 0.05 | 0.01 | G | 1.554 | 0.213 | 3.646 |
| 161 | 13395 | G | 0.02 | 0.01 | A | 0.07953 | 0.778 | 1.414 |
| 162 | 13563 | G | 0.01 | 0.01 | A | 0.0614 | 0.804 | 0.7041 |
| 163 | 13635 | C | 0.05 | 0.03 | T | 0.4969 | 0.481 | 1.809 |
| 164 | 13656 | C | 0.01 | 0.10 | T | 7.349 | 0.007 | 0.09184 |
| 165 | 13674 | C | 0.01 | 0.01 | T | 0.0614 | 0.804 | 0.7041 |
| 166 | 13708 | A | 0.09 | 0.07 | G | 0.2048 | 0.651 | 1.3 |
| 167 | 13731 | G | 0.01 | 0.01 | A | 0.0614 | 0.804 | 0.7041 |
| 168 | 13759 | A | 0.01 | 0.03 | G | 0.8023 | 0.370 | 0.3469 |
| 169 | 13780 | G | 0.01 | 0.01 | A | 0.06505 | 0.799 | 0.697 |
| 170 | 13818 | C | 0.01 | 0.01 | T | 0.0614 | 0.804 | 0.7041 |
| 171 | 13928 | C | 0.03 | 0.03 | G | 0.004283 | 0.948 | 1.062 |
| 172 | 13966 | G | 0.01 | 0.03 | A | 0.8023 | 0.370 | 0.3469 |
| 173 | 13971 | T | 0.04 | 0.03 | C | 0.1677 | 0.682 | 1.432 |
| 174 | 14040 | A | 0.06 | 0.04 | G | 0.2562 | 0.613 | 1.441 |
| 175 | 14128 | G | 0.01 | 0.04 | A | 1.904 | 0.168 | 0.2279 |
| 176 | 14131 | T | 0.01 | 0.01 | C | 0.0614 | 0.804 | 0.7041 |
| 177 | 14323 | A | 0.05 | 0.06 | G | 0.04693 | 0.829 | 0.8611 |
| 178 | 14544 | A | 0.04 | 0.03 | G | 0.1512 | 0.697 | 1.407 |
| 179 | 14569 | A | 0.05 | 0.02 | G | 1.525 | 0.217 | 3.611 |
| 180 | 14783 | T | 0.19 | 0.26 | C | 1.062 | 0.303 | 0.6738 |
| 181 | 14905 | A | 0.01 | 0.01 | G | 0.0464 | 0.829 | 0.7368 |
| 182 | 14935 | C | 0.01 | 0.01 | T | 0.0464 | 0.829 | 0.7368 |
| 183 | 14990 | T | 0.03 | 0.04 | C | 0.1427 | 0.706 | 0.7312 |
| 184 | 15043 | G | 0.38 | 0.34 | A | 0.3745 | 0.541 | 1.22 |
| 185 | 15049 | T | 0.03 | 0.06 | C | 0.6397 | 0.424 | 0.5403 |
| 186 | 15061 | G | 0.02 | 0.01 | A | 0.1054 | 0.746 | 1.489 |
| 187 | 15097 | C | 0.03 | 0.01 | T | 0.5144 | 0.473 | 2.258 |
| 188 | 15148 | A | 0.04 | 0.01 | G | 1.069 | 0.301 | 3.043 |
| 189 | 15218 | G | 0.01 | 0.03 | A | 0.7291 | 0.393 | 0.3632 |
| 190 | 15251 | T | 0.03 | 0.01 | C | 0.5144 | 0.473 | 2.258 |
| 191 | 15262 | C | 0.02 | 0.03 | T | 0.08751 | 0.767 | 0.7419 |
| 192 | 15301 | G | 0.37 | 0.32 | A | 0.4489 | 0.503 | 1.245 |
| 193 | 15314 | A | 0.01 | 0.03 | G | 0.7291 | 0.393 | 0.3632 |
| 194 | 15385 | T | 0.05 | 0.01 | C | 1.701 | 0.192 | 3.846 |
| 195 | 15431 | A | 0.06 | 0.08 | G | 0.2964 | 0.586 | 0.7222 |
| 196 | 15452 | A | 0.01 | 0.01 | C | 0.0464 | 0.829 | 0.7368 |
| 197 | 15601 | C | 0.01 | 0.03 | T | 0.8427 | 0.359 | 0.3385 |
| 198 | 15670 | C | 0.01 | 0.04 | T | 1.904 | 0.168 | 0.2279 |
| 199 | 15712 | G | 0.01 | 0.01 | A | 0.0701 | 0.791 | 0.6875 |
| 200 | 15784 | C | 0.01 | 0.03 | T | 0.8023 | 0.370 | 0.3469 |
| 201 | 15884 | C | 0.01 | 0.01 | G | 0.0701 | 0.791 | 0.6875 |
| 202 | 15908 | C | 0.02 | 0.03 | T | 0.1419 | 0.706 | 0.6842 |
| 203 | 15924 | G | 0.01 | 0.04 | A | 1.904 | 0.168 | 0.2279 |
| 204 | 15927 | A | 0.02 | 0.01 | G | 0.07152 | 0.789 | 1.389 |
| 205 | 15928 | A | 0.05 | 0.04 | G | 0.05319 | 0.818 | 1.188 |
| 206 | 15930 | A | 0.03 | 0.06 | G | 0.744 | 0.388 | 0.5156 |
| 207 | 16037 | G | 0.01 | 0.01 | A | 0.0701 | 0.791 | 0.6875 |
| 208 | 16051 | G | 0.09 | 0.15 | A | 1.234 | 0.267 | 0.583 |
| 209 | 16086 | A | 0.03 | 0.01 | T | 0.4265 | 0.514 | 2.106 |
| 210 | 16093 | C | 0.08 | 0.03 | T | 1.917 | 0.166 | 2.921 |
| 211 | 16104 | T | 0.03 | 0.01 | C | 0.4265 | 0.514 | 2.106 |
| 212 | 16111 | T | 0.02 | 0.03 | C | 0.1419 | 0.706 | 0.6842 |
| 213 | 16126 | C | 0.07 | 0.10 | T | 0.5299 | 0.467 | 0.6667 |
| 214 | 16129 | A | 0.25 | 0.11 | G | 4.987 | 0.026 | 2.618 |
| 215 | 16140 | C | 0.01 | 0.04 | T | 1.978 | 0.160 | 0.2222 |
| 216 | 16145 | A | 0.09 | 0.01 | G | 4.325 | 0.038 | 6.9 |
| 217 | 16162 | A | 0.02 | 0.07 | G | 2.71 | 0.100 | 0.268 |
| 218 | 16172 | C | 0.04 | 0.03 | T | 0.1458 | 0.703 | 1.398 |
| 219 | 16173 | T | 0.01 | 0.01 | C | 0.0701 | 0.791 | 0.6875 |
| 220 | 16183 | C | 0.06 | 0.03 | A | 0.9331 | 0.334 | 2.194 |
| 221 | 16184 | T | 0.03 | 0.03 | C | 0.001555 | 0.969 | 1.037 |
| 222 | 16185 | T | 0.02 | 0.01 | C | 0.07152 | 0.789 | 1.389 |
| 223 | 16189 | C | 0.08 | 0.01 | T | 3.486 | 0.062 | 5.933 |
| 224 | 16194 | C | 0.06 | 0.01 | A | 2.136 | 0.144 | 4.352 |
| 225 | 16206 | C | 0.03 | 0.03 | A | 0.001555 | 0.969 | 1.037 |
| 226 | 16209 | C | 0.02 | 0.06 | T | 1.717 | 0.190 | 0.3316 |
| 227 | 16223 | C | 0.28 | 0.22 | T | 0.6172 | 0.432 | 1.337 |
| 228 | 16230 | G | 0.02 | 0.04 | A | 0.7824 | 0.376 | 0.4491 |
| 229 | 16234 | T | 0.04 | 0.04 | C | 0.01215 | 0.912 | 0.9176 |
| 230 | 16239 | T | 0.03 | 0.07 | C | 1.631 | 0.202 | 0.3957 |
| 231 | 16249 | C | 0.01 | 0.03 | T | 0.8427 | 0.359 | 0.3385 |
| 232 | 16255 | C | 0.01 | 0.01 | G | 0.05993 | 0.807 | 0.7071 |
| 233 | 16260 | T | 0.05 | 0.01 | C | 1.582 | 0.209 | 3.684 |
| 234 | 16261 | T | 0.13 | 0.01 | C | 7.422 | 0.006 | 10.46 |
| 235 | 16263 | T | 0.02 | 0.01 | C | 0.0843 | 0.772 | 1.429 |
| 236 | 16264 | T | 0.03 | 0.01 | C | 0.4604 | 0.497 | 2.165 |
| 237 | 16266 | T | 0.05 | 0.04 | C | 0.05587 | 0.813 | 1.193 |
| 238 | 16270 | T | 0.01 | 0.06 | C | 3.141 | 0.076 | 0.1692 |
| 239 | 16274 | A | 0.05 | 0.04 | G | 0.05587 | 0.813 | 1.193 |
| 240 | 16290 | T | 0.01 | 0.01 | C | 0.06349 | 0.801 | 0.7 |
| 241 | 16294 | T | 0.05 | 0.06 | C | 0.03926 | 0.843 | 0.8724 |
| 242 | 16298 | C | 0.01 | 0.01 | T | 0.06349 | 0.801 | 0.7 |
| 243 | 16302 | G | 0.01 | 0.01 | A | 0.06349 | 0.801 | 0.7 |
| 244 | 16304 | C | 0.12 | 0.08 | T | 0.5236 | 0.469 | 1.461 |
| 245 | 16309 | G | 0.02 | 0.01 | A | 0.07953 | 0.778 | 1.414 |
| 246 | 16319 | A | 0.05 | 0.08 | G | 0.8533 | 0.356 | 0.5642 |
| 247 | 16320 | T | 0.01 | 0.01 | C | 0.06349 | 0.801 | 0.7 |
| 248 | 16325 | C | 0.04 | 0.04 | T | 0.007497 | 0.931 | 0.9347 |
| 249 | 16356 | C | 0.08 | 0.04 | T | 0.9511 | 0.329 | 1.95 |
| 250 | 16362 | C | 0.11 | 0.08 | T | 0.2788 | 0.598 | 1.324 |
| 251 | 16381 | C | 0.03 | 0.01 | T | 0.4477 | 0.503 | 2.143 |
| 252 | 16390 | A | 0.06 | 0.06 | G | 0.007166 | 0.933 | 1.058 |
| 253 | 16391 | A | 0.02 | 0.03 | G | 0.1285 | 0.720 | 0.697 |
| 254 | 16527 | T | 0.02 | 0.01 | C | 0.07953 | 0.778 | 1.414 |

MAF: Minor Allele Frquency; CHISQ: Chi-sqaure; OR: Odd’s Ratio
