# Supplementary material for: Mitochondrial Genome Analysis of Primary Open Angle Glaucoma Patients
Source: PLoS One. 2013 Aug 5;8(8):e70760. doi: 10.1371/journal.pone.0070760 (PMC3733777; doi:10.1371/journal.pone.0070760)
Supplement: Table S10 — List of haplogroups of individual patients. (DOCX) [file pone.0070760.s010.docx]

**Table S10: List of haplogroups of individual patients**

| **Sl No** | **Patient code** | **Haplogroup** |  | **Sl No** | **Patient code** | **Haplogroup** |
| --- | --- | --- | --- | --- | --- | --- |
| 1 | GL425 | L3 |  | 52 | GL610 | R |
| 2 | GL434 | M |  | 53 | GL611 | R |
| 3 | GL437 | M |  | 54 | GL612 | M |
| 4 | GL439 | L3 |  | 55 | GL613 | L3 |
| 5 | GL441 | M |  | 56 | GL614 | M |
| 6 | GL442 | M |  | 57 | GL617 | M |
| 7 | GL444 | M |  | 58 | GL619 | M |
| 8 | GL448 | M |  | 59 | GL620 | M |
| 9 | GL466 | L3 |  | 60 | GL625 | M |
| 10 | GL473 | C |  | 61 | GL626 | M |
| 11 | GL477 | M |  | 62 | GL627 | M |
| 12 | GL479 | R |  | 63 | GL630 | NA |
| 13 | GL480 | D |  | 64 | GL631 | L3 |
| 14 | GL481 | U |  | 65 | GL632 | R |
| 15 | GL497 | M |  | 66 | GL633 | M |
| 16 | GL500 | M |  | 67 | GL634 | L3 |
| 17 | GL512 | M |  | 68 | GL635 | M |
| 18 | GL520 | M |  | 69 | GL638 | M |
| 19 | GL521 | L3 |  | 70 | GL641 | M |
| 20 | GL522 | M |  | 71 | GL642 | L3 |
| 21 | GL523 | M |  | 72 | GL648 | NA |
| 22 | GL525 | M |  | 73 | GL649 | M |
| 23 | GL526 | R |  | 74 | GL650 | M |
| 24 | GL529 | M |  | 75 | GL655 | M |
| 25 | GL538 | M |  | 76 | GL658 | R |
| 26 | GL539 | R |  | 77 | GL663 | R |
| 27 | GL540 | U |  | 78 | GL665 | U |
| 28 | GL541 | M |  | 79 | GL667 | M |
| 29 | GL546 | M |  | 80 | GL668 | R |
| 30 | GL550 | M |  | 81 | GL675 | M |
| 31 | GL552 | R |  | 82 | GL684 | R |
| 32 | GL558 | L3 |  | 83 | GL686 | R |
| 33 | GL559 | **N** |  | 84 | GL690 | U |
| 34 | GL561 | **M** |  | 85 | GL691 | M |
| 35 | GL562 | NA |  | 86 | GL692 | M |
| 36 | GL563 | **U** |  | 87 | GL696 | M |
| 37 | GL565 | M |  | 88 | GL700 | M |
| 38 | GL569 | NA |  | 89 | GL701 | M |
| 39 | GL571 | R |  | 90 | GL703 | N |
| 40 | GL580 | R |  | 91 | GL706 | M |
| 41 | GL587 | M |  | 92 | GL708 | R |
| 42 | GL595 | M |  | 93 | GL709 | M |
| 43 | GL597 | M |  | 94 | GL711 | M |
| 44 | GL598 | M |  | 95 | GL851 | M |
| 45 | GL600 | M |  | 96 | GL876 | R |
| 46 | GL601 | U |  | 97 | GL903 | M |
| 47 | GL602 | U |  | 98 | GL907 | U |
| 48 | GL603 | M |  | 99 | GL911 | R |
| 49 | GL604 | M |  | 100 | GL913 | D |
| 50 | GL607 | M |  | 101 | GL934 | M |
| 51 | GL609 | R |  |  |  |  |
