# Supplementary material for: Mitochondrial Genome Analysis of Primary Open Angle Glaucoma Patients
Source: PLoS One. 2013 Aug 5;8(8):e70760. doi: 10.1371/journal.pone.0070760 (PMC3733777; doi:10.1371/journal.pone.0070760)
Supplement: Table S11 — List of haplogroups of individual controls. (DOCX) [file pone.0070760.s011.docx]

**Table S11: List of haplogroups of individual controls**

| **Sl No** | **Control code** | **Haplogroup** |  | **Sl No** | **Control code** | **Haplogroup** |
| --- | --- | --- | --- | --- | --- | --- |
| 1 | GLC1 | L3 |  | 37 | GLC57 | M |
| 2 | GLC2 | M |  | 38 | GLC58 | M |
| 3 | GLC3 | M |  | 39 | GLC60 | M |
| 4 | GLC4 | M |  | 40 | GLC61 | M |
| 5 | GLC5 | M |  | 41 | GLC62 | M |
| 6 | GLC6 | M |  | 42 | GLC63 | M |
| 7 | GLC10 | M |  | 43 | GLC64 | R |
| 8 | GLC13 | R |  | 44 | GLC65 | M |
| 9 | GLC16 | U |  | 45 | GLC66 | M |
| 10 | GLC17 | M |  | 46 | GLC67 | M |
| 11 | GLC20 | M |  | 47 | GLC68 | M |
| 12 | GLC21 | M |  | 48 | GLC69 | N |
| 13 | GLC22 | M |  | 49 | GLC71 | M |
| 14 | GLC23 | M |  | 50 | GLC73 | M |
| 15 | GLC24 | M |  | 51 | GLC74 | U |
| 16 | GLC25 | U |  | 52 | GLC75 | M |
| 17 | GLC26 | M |  | 53 | GLC76 | M |
| 18 | GLC27 | R |  | 54 | GLC78 | M |
| 19 | GLC29 | M |  | 55 | GLC80 | NA |
| 20 | GLC30 | M |  | 56 | GLC81 | M |
| 21 | GLC31 | M |  | 57 | GLC82 | M |
| 22 | GLC32 | M |  | 58 | GLC84 | M |
| 23 | GLC34 | M |  | 59 | GLC85 | L3 |
| 24 | GLC35 | M |  | 60 | GLC86 | M |
| 25 | GLC38 | M |  | 61 | GLC87 | M |
| 26 | GLC40 | M |  | 62 | GLC88 | M |
| 27 | GLC41 | M |  | 63 | GLC89 | M |
| 28 | GLC42 | U |  | 64 | GLC91 | U |
| 29 | GLC43 | L3 |  | 65 | GLC92 | N |
| 30 | GLC44 | M |  | 66 | GRC1 | U |
| 31 | GLC49 | M |  | 67 | GRC3 | L3 |
| 32 | GLC50 | R |  | 68 | GRC13 | U |
| 33 | GLC52 | U |  | 69 | GRC14 | M |
| 34 | GLC53 | U |  | 70 | GRC17 | R |
| 35 | GLC55 | M |  | 71 | GRC20 | L3 |
| 36 | GLC56 | M |  |  |  |  |
